# Use of HC2 to triage women with borderline and mild dyskaryosis in the UK

**DOI:** 10.1038/bjc.2011.351

**Published:** 2011-09-27

**Authors:** M Arbyn, J Roelens, P Martin-Hirsch, S Leeson, N Wentzensen

**Affiliations:** 1 Unit of Cancer Epidemiology, Scientific Institute of Public Health, Brussels, Belgium; 2 Department of Obstetrics and Gynaecology, Central Lancashire Teaching Hospitals, Preston, UK; 3 Department of Obstetrics and Gynaecology, Betsi Cadwaladr University Health Board, North Wales, UK; 4 Division of Cancer Epidemiology and Genetics, National Cancer Institute, NIH, DHHS, Bethesda, USA

To investigate the feasibility and efficiency of a nationwide rollout of human papillomavirus (HPV)-based triage for cervical cytology results of borderline (BD) or mild dyskaryosis (MD) in the UK, a study at six sentinel sites including more than 10 000 women aged 25–64 was conducted in a real life setting (Kelly *et al*, 2011). The applied triage test was the Hybrid Capture-2 assay (HC2, Qiagen, GmbH, Hilden, Germany), which detects DNA of 13 high-risk (hr) HPV types. The cut-off chosen for a positive HPV test was ⩾2 RLU/PC (relative light units/positive control), which is lower than what was used in a previous pilot study (⩾3 RLU/PC), but higher than what is widely used in Europe and the US (⩾1 RLU/PC). All HPV-positive women were referred for colposcopy involving biopsies from visually abnormal areas of the cervical epithelium. HPV-negative women and HPV-positive women with negative colposcopy or histology findings were returned to the usual screening schedule. A histological result of CIN1 (cervical intraepithelial neoplasia of grade 1) resulted in a follow-up cytology 12 months later, whereas CIN2 or worse lesions (CIN2+) were treated. Outcomes were the referral rate to colposcopy (or rate of HPV-positivity) and the yield of underlying CIN2+ or CIN3+ among referred women (positive predictive value (PPV)). To evaluate the heterogeneity of HPV prevalence and PPV for cervical disease, several covariates were addressed, including age of the woman, type of liquid-cytology (SurePath (BD, TriPath Imaging inc., Burlington, NC, USA) or ThinPrep (Hologic Inc., Marlborough, MA, USA)) and laboratory site.

Below, we summarize the results of the six sentinel studies and include them into previously published meta-analyses on the same topic (Arbyn *et al*, 2005; Arbyn *et al*, 2006; Arbyn *et al*, 2009).

## Description of the UK pilot data

**Figure 1:**
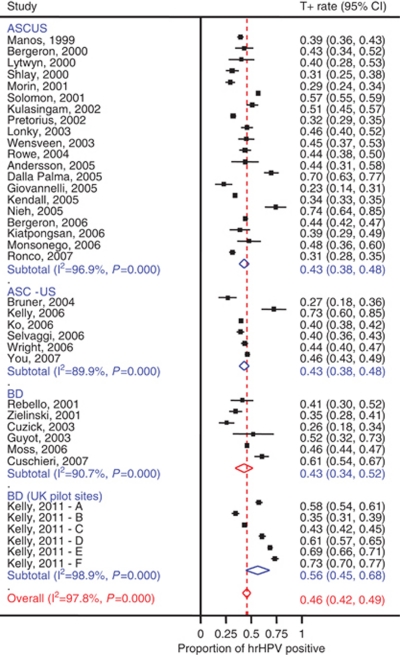
Proportion of women with ASCUS or borderline dyskaryosis (BD) that have a positive Hybrid Capture II test, derived from a previously published meta-analysis completed with data from the UK Pilot Study. The consistency index *I*^*2*^ expresses the proportion of total variation due to inter-study heterogeneity; the *P* value corresponds with a χ^2^ test for inter-study heterogeneity.

**Figure 2:**
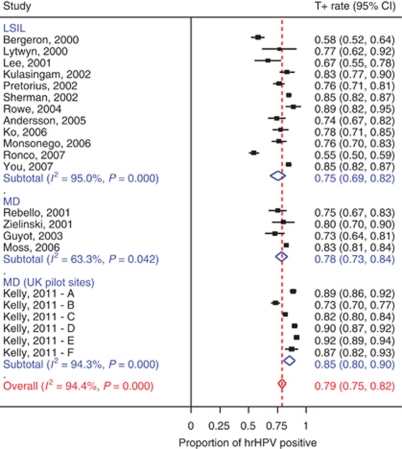
Proportion of women with LSIL or mild dyskaryosis (MD) that have a positive Hybrid Capture II test, derived from a previously published meta-analysis completed with data from the UK Pilot Study.

**Figure 3:**
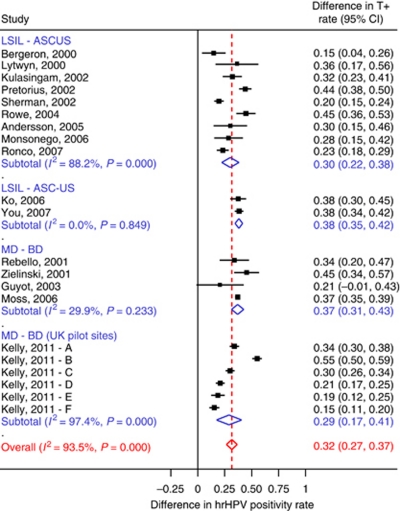
Difference in high-risk HPV test positivity rate between LSIL/mild dyskaryosis (MD) and ASCUS/borderline dyskaryosis (BD) cases.

The hrHPV positivity rates varied widely among sites: between 35% and 73% in BD and between 73 and 92% in MD. The pooled hrHPV rates were significantly higher in BD (56%, 95% CI: 45–68%) compared to MD (85%, 95% CI: 80–90%). The pooled estimates of test positivity (computed using a random effect meta-analytical procedure) are slightly different from those reported and computed by summation of the data from the six sites. The differences between BD and MD were consistent among the sites (see Figures 1, 2 and 3). Moreover, hrHPV rates decreased significantly by age, in particular in women with BD (69% in age group 25–34 *vs* 31% in age group 50–64), whereas the age trend was less pronounced in women with MD (89% in age group 25–34 and 67% in age group 50–64). This finding is expected, since BD is a heterogeneous group of HPV-related changes and non-HPV related changes related to epithelial repair or inflammation, while MD is a specific sign of HPV effects (Zuna *et al*, 2005). Adjustment for age did not change substantially the differences in hrHPV rates between BD and MD and among sites.

The PPVs for underlying high-grade disease were also highly variable between sites, ranging from 9–22% in BD for the outcome of CIN2+, 3–12% in BD for CIN3+, 9–30% in MD for CIN2+ and 3–15% in MD for CIN3+. The PPVs were highly correlated at the level of the sites (*ρ* Spearman ⩾0.88) with high values in sites E and F, low values in site D. Remarkably, site D also had a very high CIN2/CIN3+ ratio, suggesting differences in the interpretation of histology results between the sites. PPVs decreased also significantly by age. The overall PPV for CIN2+ was similar in BD and MD (16 and 17%, respectively, ratio=0.93, 95% CI: 0.83–1.04). However, for CIN3+, the PPV was significantly higher in BD (6.7%) compared to MD (5.4%): ratio 1.25, 95% CI: 1.02–1.53.

## Comparison with previous meta-analyses

The forest plots in Figures 1 and 2 display the hrHPV positivity rates among women with BD/ASCUS (atypical squamous cells of uncertain significance) and MD/LSIL (low-grade squamous intraepithelial lesions), respectively. The hrHPV positivity rates observed in the six British pilot sites were among the highest reported in the literature on HPV triage of minor cytological abnormalities (Figures 1 and 2) and the rate observed in women of site E (92%) was the highest of those reported in women with LSIL or MD. The hrHPV rates at the British pilot sites would even be a few percentages higher if the RLU>1 cutoff would have been applied as in other studies. In ALTS, 4.5% of women with ASCUS and 3.5% of women with LSIL were in the range of 1–2 RLU/PC (Sherman *et al*, 2002). The study further confirms the consistent difference in high-risk HPV positivity rate between BD/ASCUS and MD/LSIL: on average 33% (95% CI: 29–37%). This difference was significantly different from zero in nearly all the separate studies of the meta-analysis (see Figure 3), including the six separate pilot laboratories, each age group and preparation method (SurePath or ThinPrep).

**Figure 4:**
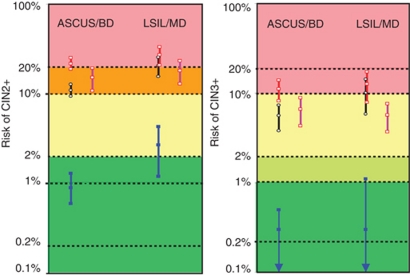
Absolute risk of underlying or incipient CIN2+ (left) or CIN3+ (right) in women with ASCUS or borderline dyskaryosis (BD) and LSIL or mild dyskaryosis (MD), according to the result of the HC2 HPV assay, derived from a meta-analysis (Arbyn *et al*, 2009, JCMM). Black: independent of HPV test=prevalence of disease; Blue: when hrHPV test is negative (=1-NPV); Red: when hrHPV test is positive (=PPV). Coloured lines contain the pooled mean and its 95% CI. For comparison the PPV of HC2 test observed in the British Pilot study (Kelly *et al*, 2011) are put at right (in purple) of the PPV from the meta-analysis.

The purpose of a triaging is to increase the probability of finding relevant disease in those with a positive triage test result (=PPV) justifying further diagnostic and/or therapeutic procedures and/or to downgrade the probability of finding such disease in those with a negative test (cNPV (complement of the negative predictive value)=1-NPV). The risk diagram, in Figure 4, provides a clinically useful presentation of this concept (Castle *et al*, 2007). In the green zones (risk CIN2+ <2%, risk CIN3+<1%), one can accept referring a patient to the normal screening schedule. In the orange zone (risk CIN2+ between 10 and 20%) or red zone (risk CIN2+ >20% or risk CIN3+>10%), referral to colposcopy is usually considered as good practice. The risk diagram contains the estimates of the following triage performance parameters and their 95% CIs derived from a meta-analysis: cNPV (blue lines), PPV (red lines) completed with the pooled PPV from the British pilot study (purple lines). The meta-analytically pooled prevalence of disease (=pretest probability) is presented as a black line). In all cytology groups (ASCUS/BD and LSIL/MD) and outcomes (CIN2+ or 3+), the cNPV estimates were situated in the safe green zone, except in women with LSIL/MD, where the average risk for CIN2+ was 2.7%, (in the yellow zone). In ASCUS triage, conditions for clinical utility were generally fulfilled: the PPV was obviously higher than the pretest probability and the red PPV lines were always in orange/red zones and clearly separated from the black (pretest) lines. However, in LSIL triage, the pretest probability is hardly different from the PPV (red lines overlapping with black lines which are already in the orange/red zone). In the British pilot study, only the PPV could be assessed. The PPV for CIN2+ (purple lines) was >10% (orange risk zone) in both BD and MD. However, in MD, the average pretest probability of CIN2+ was already 24% in four other British triage studies, indicating only a marginal triage gain in this cytological category (Rebello *et al*, 2001; Guyot *et al*, 2003; Szarewski *et al*, 2008; Cuschieri *et al*, 2010). For the outcome CIN3+, the PPV of HC2 in MD was only 5% which was significantly lower than in BD.

### Conclusions

The findings of the pilot study reported in this issue of the British Journal of Cancer are in line with findings from meta-analyses which confirm the utility of HC2 to triage women with borderline cervical lesions regardless of age. However, nearly all women with MD were HC2 positive, at the exception of those aging 50 or older, undermining its general use to discriminate women at risk of having a cancer precursor. HC2 could become cost-effective only *in situations* where referral to colposcopy and further management are very expensive and the triage test is very cheap, leading to savings by a small reduction of colposcopy referrals. Currently, in Europe and the USA, HPV triage of MD/LSIL is not considered cost-effective and referral to colposcopy or repeat cytology are recommended options (Solomon *et al*, 2009; Jordan *et al*, 2008). Recently, more disease-specific triage options have been emerging, such as HPV16/18 genotyping, RNA testing for a limited number of hr HPV types or p16. These biomarkers may have high enough sensitivity to identify women at highest risk of CIN2 or greater, while having improved specificity compared to HPV DNA testing to further reduce referral of women with MD/LSIL to colposcopy. These approaches could lead to optimising efficiency of diagnostic services and avoiding over-treatment. Further, it would reduce the problem of drop-outs from follow-up which is typical for delayed triage algorithms. However, depending on how long a negative biomarker test can reassure against developing high grade CIN, women with minor cervical abnormalities being negative for disease specific triage tests may be required to return after a year for safety reasons. Further research is needed to identify triage tests usable in LSIL or BD that combine both high PPV and low cNPV.

Another take-home message from the study by Kelly *et al* is the apparent heterogeneity of cervical cytology interpretation, even within a centralized healthcare system. Significant deviations of HPV prevalence from the summary estimates provided in the meta-analysis figures (Figures 1 and 2) can indicate systematic over- or undercalling of cervical cytology. Thus, HPV testing of representative cytology specimens at individual sites could serve as a simple and objective quality control measure (Arbyn *et al*, 2009).
